# A new calmanostracan crustacean species from the Cretaceous Yixian Formation and a simple approach for differentiating fossil tadpole shrimps and their relatives

**DOI:** 10.1186/s40851-019-0136-0

**Published:** 2019-06-18

**Authors:** Philipp Wagner, Joachim T. Haug, Carolin Haug

**Affiliations:** 1 Department of Biology II, LMU Munich, Biocenter, Großhaderner Str. 2, 82152, Planegg-Martinsried, Germany; 2 GeoBio-Center der LMU München, Richard-Wagner-Str. 10, 80333 Munich, Germany

**Keywords:** Eucrustacea, Calmanostraca, Notostraca, Kazacharthra, Phylogeny, Morphospace, Species delineation, Yixian Formation, Jehol Group, Cretaceous

## Abstract

**Background:**

Calmanostraca is a group of branchiopod eucrustaceans, with *Triops cancriformis* and *Lepidurus apus* as most prominent representatives. Both are regularly addressed with the inaccurate tag “living fossil”, suggesting that the morphology has remained stable for several millions of years. Yet, *T. cancriformis* and *L. apus* represent only a fraction of the morphological diversity occurring in Calmanostraca, comprising the two groups Notostraca and Kazacharthra. Notostracans, commonly called tadpole shrimps, comprise the two groups *Lepidurus* and *Triops* with their elongated and rather narrow (in dorsal view) head shields*.* Kazacharthrans are exclusively fossil calmanostracans with broad and rather short shields, known from the Jurassic and Triassic period. One formation where fossil calmanostracans have been found is the Yixian Formation of northeastern China (Lower Cretaceous, 125–121 million years). It is part of the Jehol Group, an ecosystem known for its exceptionally well-preserved fossils, including vertebrates and plants, but also diverse arthropods. Two calmanostracan species have to date been described from the Yixian Formation, *Jeholops hongi* and *Chenops yixianensis*.

**Results:**

We describe here a new calmanostracan crustacean from the Yixian Formation, *Notostraca oleseni*, and additionally a simple tool using a morphospace analysis to delineate different species. Measurements characterising the shield and trunk proportions of different calmanostracan species were performed, data were size-corrected, and used for this morphospace analysis to compare the different morphologies. As sclerotised body parts are more likely to be preserved in fossils than soft tissue, shields and parts of the trunk are in many cases the only morphological structures available for study. Therefore, the present analysis represents a simple tool for distinguishing between different species, as well as allowing the inclusion of specimens that are only preserved fragmentarily. Additionally, it provides a tool to demarcate the kazacharthran-like specimen described, but not formally named, by Wagner et al. (Paleontol Res. 22:57–63, 2018). Hence, we amended the description and name the species *Calmanostraca hassbergella*.

**Conclusion:**

Our results indicate a large diversity in shield and trunk morphology in calmanostracans, in contrast to their often claimed highly conserved and uniform morphology. Especially extinct forms such as *Notostraca oleseni* add up to this result and point to the species richness and morphological diversity within Calmanostraca.

## Background

Tadpole shrimps are notostracan crustaceans that are well known for two different reasons: 1) They can easily be bred at home and therefore are very popular as a kind of “pet”. 2) They are often referred to as “living fossils”, which inaccurately suggests that their morphology has barely changed for some hundreds of million years (e.g. [16, 28, 29, 38]; but see [57] for a critical discussion).

Notostracan crustaceans occur in freshwater habitats all around the world, except for Antarctica (e.g. [47]). They are specialised to life in ephemeral ponds, temporary small lakes, and pools which tend to drain seasonally. As one specialisation to these short-termed habitats they produce desiccation-resistant eggs, which are able to survive longer periods without water [9, 33, 38, 61].

Within Notostraca generally two distinct groups are distinguished, *Lepidurus* and *Triops* (although the case is slightly more complicated, see further below). Representatives of Notostraca all share a common body organization. Most prominent is a dorsal shield (formed by the head segments), which is strongly drawn out laterally and posteriorly. Dorsally an elevation with eye structures is present. The head bears five pairs of appendages: 1) small, not subdivided antennulae, 2) small antennae, 3) large mandibles guided by paragnaths (sometimes mistaken for appendages), 4) small maxillulae, and 5) small maxillae. The trunk is subdivided into three regions. The anterior trunk region features 11 serial segments, each bearing one pair of appendages (anterior thoracopods). The appendages on the first thoracic segment are modified into sensory structures, which are strongly elongated (in most representatives). The further posterior trunk region is also subdivided into segments. The segments of the middle trunk region also bear appendages (posterior thoracopods), yet in contrast to the segments of the anterior trunk region often one segment bears several appendage pairs. The segments of the posterior trunk region do not bear any appendages (this body region is commonly termed abdomen). The last trunk segment articulates to the telson, which is extended into an anal plate in representatives of *Lepidurus*, but not in those of *Triops*. In representatives of both groups the telson bears a pair of elongated, multiannulated, furcal rami (e.g. [8, 12, 27, 38, 43, 59, 63]).

Notostraca is an ingroup of Calmanostraca, a larger monophyletic group which besides Notostraca also includes its sister group, Kazacharthra (presumably; see below); the latter is a group with exclusively fossil representatives restricted to the Triassic and possibly Jurassic period (e.g. [3, 42, 44–46]). Kazacharthrans differ from notostracans, for example, in having a broader and rather flat shield and in lacking a dorsal median ridge (e.g. [41]). However, calmanostracan phylogeny seems in fact to be a little bit more complicated. Especially the phylogenetic positions of several species, such as the fossil species *Strudops goldenbergi*, *Notostraca minor* (often referred to as *Triops cancriformis minor,* or *Triops minor* in older literature, see [57] for this issue), but also extant forms such as *Lepidurus batesoni* (or “*Lepidurus*” *batesoni*, see e.g [34]) are still far from being reliably settled. Depending on the exact position of *S. goldenbergi* and *N. minor*, Notostraca could either be considered as non-monophyletic, or Notostraca could turn out to be synonymous to Calmanostraca (we currently see the latter option as more practical; also see [34] and discussion below).

While it is common to identify morphological differences qualitatively, i.e. “the shield of kazacharthrans is broader than that of notostracans,” there are also ways to express such differences more quantitatively. An example of such an approach is the construction of a morphospace. Morphospaces are multi-dimensional spaces representing the form of organisms, or a specific structure of an organism in detail, based on measurements, hence quantitative evaluations, of these structures (see e.g. [1, 15] for similar approaches). Such measurements can, in the case of calmanostracans, for example include different dimensions of the specimen, such as ‘the maximum width of the dorsal shield’ or; the width of the anterior trunk’. After corrections for overall size such measurements can be plotted as a morphospace which yields quantifiable information about the overall body shape.

Here we describe a new fossil calmanostracan crustacean specimen from the Yixian Formation (Lower Cretaceous, 125–121 million years, China). So far, two calmanostracan species are known from the Yixian Formation, *Jeholops hongi* and *Chenops yixianensis* [24]. We provide a simple morphospace analysis including the new specimen and different species of Calmanostraca and discuss aspects of the overall shape diversity of calmanostracan crustaceans.

## Materials and methods

### Material and geological setting

The center of the study is a single specimen purchased during the fossil fair in Leinfelden-Echterdingen in March 2017. According to information provided by the seller, the fossil was collected from the Yixian Formation near Beipiao, Liaoning in the northeast of China. Comparison to specimens from this formation (especially details of the matrix) support this information.

The Yixian Formation is part of the Jehol Group, which comprises a preserved ecosystem, dominated by lakes and wetlands, but also periodical volcanic eruptions. The volcanic ash falls seem to have quickly buried the organisms under anoxic conditions, which led to their exceptionally good preservation [65]. The formation is famous for vertebrates and plants, but also a diverse arthropod fauna is known from there [64]. This also includes *Jeholops hongi* and *Chenops yixianensis*, two notostracans described by Hegna & Ren [24]. There has been a debate about the age of the Yixian Formation, whether it was deposited in the Late Jurassic or Early Cretaceous age (see discussion in [4]). Radiometric dating suggested a deposition in the Barremian, some 125–121 million years ago [53]. The Yixian Formation is followed by the slightly younger Jiufotang Formation (120–110 million years ago) [23, 65]. The herein described specimen will be stored at the Staatliches Museum für Naturkunde Stuttgart (Löwentormuseum) under the repository number SMNS 70488.

### Documentation methods

The specimen was photographed using a Canon EOS Rebel T3i equipped with a Canon macro lens MP-E 65mm. For illumination two Speedlite YN560-II flashes were used. To reduce reflections to a minimum the light was cross-polarized (e.g., [6, 20, 21, 30, 50]). Due to the limited depth of field of one single image, it was necessary to take several images in different focus layers (image stacks), as the specimen shows a slight three-dimensional preservation. These stacks were fused into one sharp image using the freeware Combine ZM. Due to the limited field of view it was not possible to fit the specimen into one single image with sufficient resolution. Therefore, adjacent sharp images were stitched together using the photomerge function of Adobe Photoshop CS4 (see [19]). Adobe Photoshop Elements 15 was used to optimize brightness and contrast. Additionally, the filter “unsharp mask” was used to reduce blur, resulting in high resolution images.

### Measurements

We measured three dimensions to characterize the coarse overall body shape: 1) shield length, from the anterior tip of the shield to a constructed line between the lateral tips of the posterior notch, 2) shield width, at the widest point of the shield, and 3) the trunk width, again at the widest point. Measurements were performed using Adobe Acrobat Reader DC.

In addition to the specimen described here, measurements were also performed on published images and additional drawings of different exemplary calmanostracan species, fossil and extant (Table 1).

**Table 1:** Measurements performed on published data. Table gives publication and respective figure within the paper used for measurements.

Species	Number of specimens measured	Publication	Figures in respective publications
*Almatium elongatum*	1	[37]	Plate I 4
*Almatium gobiense*	1	[3]	Fig. 2a
*Almatium gusevi*	3	[11]	Figs. 1, 3, 5
*Almatium gusevi*	1	[45]	Plate VIII 1
*Almatium gusevi*	7	[42]	Figs. 2A1, A2, B, C and 3B, D, E
*Calmanostraca hassbergella*	1	[58]	Fig. 2
*Chenops yixianensis*	1	[24]	Fig. 1
*Jeholops hongi*	1	[24]	Fig. 4
*Lepidurus apus*	1	[9]	Fig. 2D
*Lepidurus apus*	1	[14]	Fig. 140A
*Notostraca minor*	12	[57]	Fig. 1 + additional data
*Strudops goldenbergi*	3	[34]	Figs. 2a, b and 3f
*Triops bashuensis*	1	[11]	Plate III 2
*Triops cancriformis*	1	[9]	Fig. 2c
*Triops cancriformis*	8	[57]	Fig. 2 + additional data
*Triops hanshanensis*	1	[11]	Plate II 1b

Measurements were analysed in two different ways. In general, data need to be size-corrected, otherwise the majority of results would not be able to indicate differences in form and shape of the specimen, but be mainly influenced by the body size of the specimen. Hence these data would mainly show differences in size, not in form and shape. The simplest method to eliminate size is calculating ratios. In our study, the ratio of anterior trunk width/shield width was plotted over the ratio of shield width/shield length.

However, calculating such ratios has been criticized in the past, still being size-dependent ([1, 2, 5]; but also [25]). Several alternative size correction methods have been proposed. One of these methods is the Burnaby-Size-Correction method, using the Burnaby-Back-Projection [10, 15, 31].

The Burnaby-Back-Projection uses all measurements in an n-dimensional space, creates one data point for each specimen, and assumes a growth vector for each of these points. All growth vectors point in the same direction (also see [40]), representing past and future growth stages of the specimen. These vectors are then projected onto a (n-1)-dimensional space orthogonal to the growth vectors. Therefore, a data point which was projected in this way loses its information about its growth stage, and only gives information about its location in the space and also in relation to all other points. Hence, the effect of size is removed from the data set. We used the Burnaby-Back-Projection method R-Code written by Eberle et al. [15], which is based on the R-Code by Blankers et al. [7] to perform the size correction. R packages used included readxl, FactoMineR, devtools, ggplot2, factoextra and MASS. These size-corrected data were subsequently used to calculate a principle component analysis (PCA).

### Comments on taxonomic treatment

The case presented herein represents a problematic taxonomic situation (as partly outlined above; see also [57]). Phylogeny within Calmanostraca, and especially Notostraca, is hotly debated, with molecular data suggesting a higher number of species than currently recognised; i.e. there might be numerous cryptic species [9, 26, 32, 48]. Additionally, incorporation of fossil data complicates the case even more. In the present case we do not want to complicate this situation furthermore by introducing new unnecessary supra-species group names (“genus”). To create a usable binomen, we instead suggest the usage of the name of the next reliable node above the species to be named (see [18] and references therein for such an approach).

## Results

### Description of specimen

**Fig.1:**
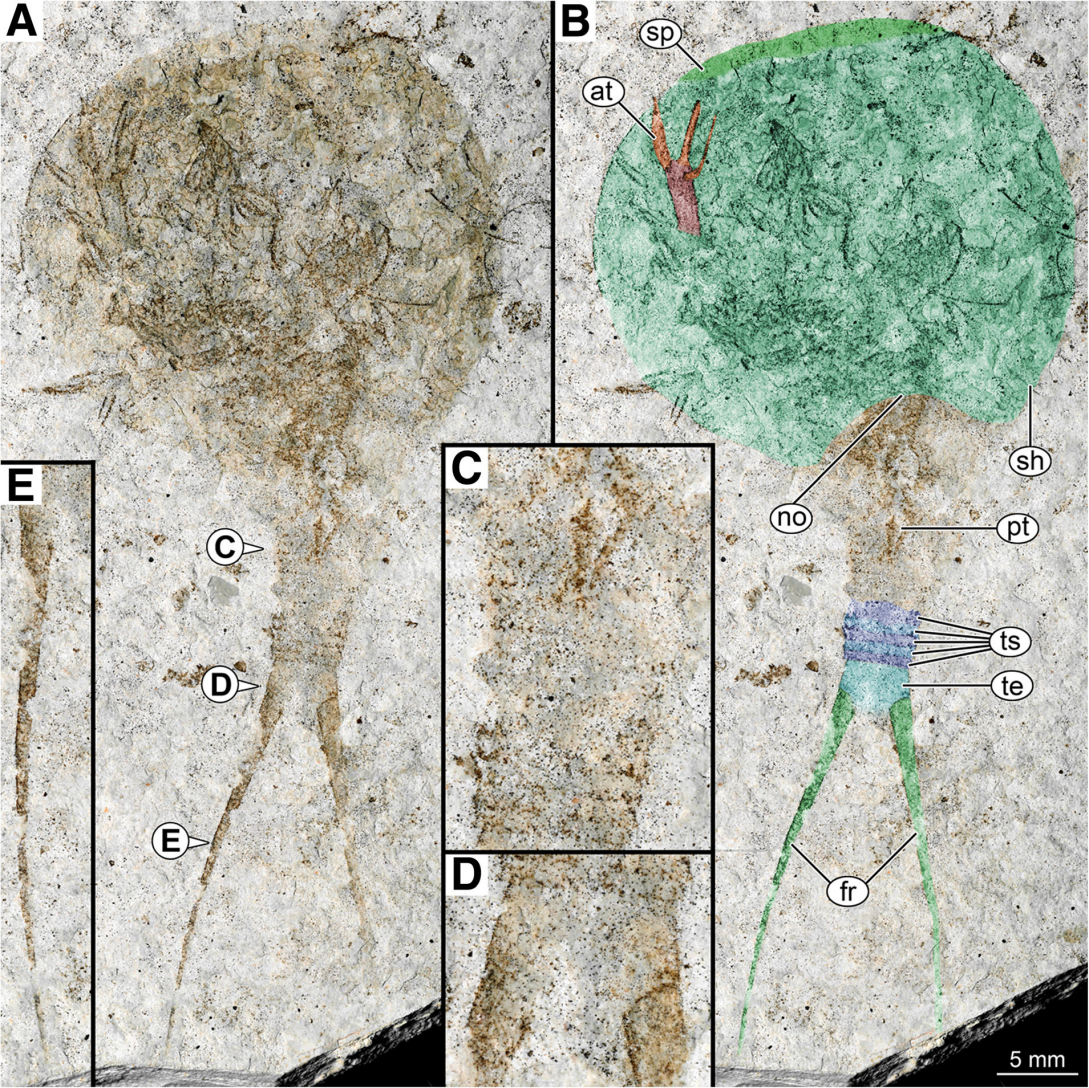
Morphology of *Notostraca oleseni* (SMNS 70488) from the Yixian Formation of north-eastern China (Lower Cretaceous, 125–121 million years). **a** *Notostraca oleseni* overview; **b** as A but with labeled structures and color markings; **c** Detail of trunk segments; **d** Detail of telson; **e** Detail of furcal rami; at, anterior thoracopod; fr, furcal rami; no, notch; pt, posterior thoracopods; sh, shield; sp, subfrontal plate; te, telson; ts, trunk segments

The fossil specimen is 68 mm long and 30 mm wide (Fig. 1a, b). Overall morphology indicates that the specimen is a representative of Calmanostraca.

**Fig. 2:**
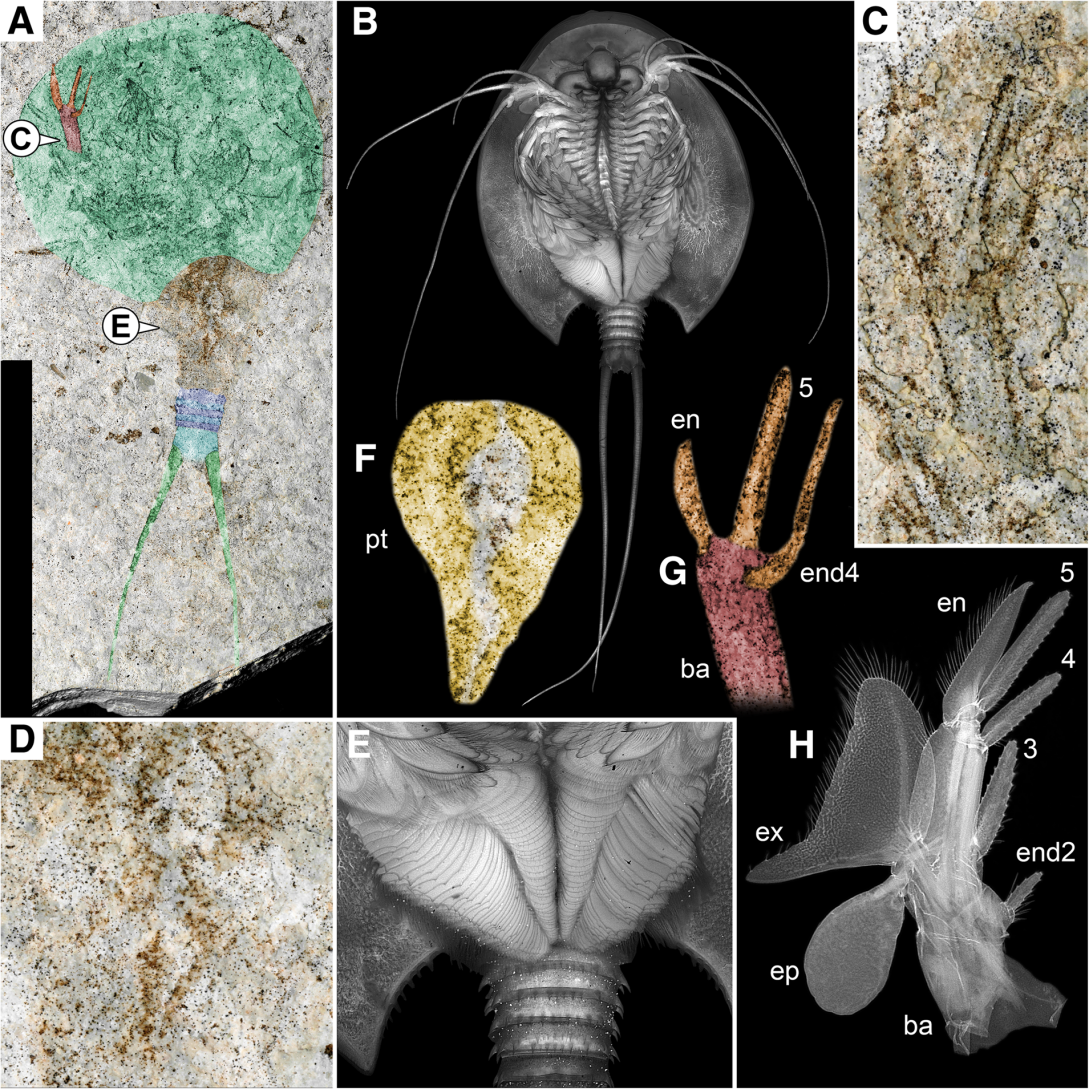
Comparison between *Notostraca oleseni* and extant *Triops cancriformis*: **a** Overview of *Notostraca oleseni*; **b** Overview of extant *Triops cancriformis*; **c** Anterior thoracopod of *Notostraca oleseni*; **d** Posterior thoracopods of *Notostraca oleseni*; **e** Posterior thoracopods of extant *Triops cancriformis*; **f** Posterior thoracopods of *Notostraca oleseni*, colour marked, detail of **d**; **g** Anterior thoracopod of *Notostraca oleseni*, colour marked, detail of **c**; **h** Anterior thoracopod of extant *Triops cancriformis*; ba, basipod; en, endopod; end2–5, endite 2–5 (endite 1 not visible on this image); ep, epipod; ex, exopod; pt, posterior thoracopods

The specimen is preserved in ventral position. The stronger sclerotized parts of the cuticle are well preserved, especially the shield is well apparent as an outline. Also, some details of the trunk are accessible. Additionally, some softer parts, such as some of the trunk appendages are more or less well preserved (Figs. 1b, c, 2 a, c, d, f, g).

The shield is prominent and dominates the overall body shape. It has a roundish shape with shield width/shield length ratio being 1.05 (Fig. 1b). The shield represents 45% of the total body length (including furcal rami, elongated, multi-annulated structures arsing from the telson; Fig. 1d, e) and 65% of the main body length (excluding furcal rami). The shield has a broad anterior doublure (“subfrontal plate” sensu [51]; Fig. 1b); any type of sculpture, such as dorsal eye structures, carinae, mandibular bulges or lateral spines are not apparent. Posteriorly the shield possesses a concave notch, with more or less rounded lateral corners (Fig. 1b). Spines in this region (as known from other calmanostracans), if present, are not visible due to the overlying trunk region. Ventrally the anterior head region, also including the mouth opening with surrounding mouthparts, is not visible. Further posteriorly several parts of the anterior trunk appendages (thoracic appendages, thoracopods) are preserved. It is not possible to identify on which distinct segment a single thoracopod attaches. A distal part of a single anterior thoracopod exhibits a good preservation (Fig. 2c, g).

This single thoracopod shows the distal part of the basipod, the endopod and two endites. The proximal part of the basipod, the lateral exopod and epipod and additional endites are not preserved (Fig. 2c, g). A thoracopod of an extant representative of the group *Triops* (Fig. 2b) is composed of a proximal basipod (with five distinct lobate endites [38], Fig. 10), bearing an endopod (strongly resembling the endites) distally, an exopod laterally and proximo-laterally to the exopod the leaf-like epipod (Fig. 2h). Following Longhurst [38] the endites preserved in the new species resemble endite 4 and 5 (Fig. 2c, g).

Posterior to the anterior thoracopods a series of posterior thoracic appendages is visible, indicating the middle trunk region (Fig. 2 d, f). Several pairs of these posterior thoracic appendages can be attached to a single segment. Individual appendages are not distinguishable, they are only recognizable in their entirety and show a characteristic slightly triangular shape which tapers posteriorly, similar to the arrangement known from extant representatives (Fig. 2e). A series of posterior trunk segments (abdominal segments) is visible. Borders between the more anterior segments are obscured. The posterior segments are well preserved, and the borders between these segments are visible (Fig. 1b, c). They are 5–6 times as wide as long. Based on these distinguishable posterior segments, we estimate a total number of about 20 segments in the posterior trunk region.

The last trunk segment articulates to the telson. The telson has a sub-trapezoidal shape in dorsal view, being slightly wider than long, with lateral notches where furcal rami attach (Fig. 1a, d). The posterior border of the telson between these attachment sites is poorly preserved (Fig. 1d). Telsonal spines are not preserved. The two furcal rami are multiannulated, elongate, and slender, tapering posteriorly. The length is about one third of the total specimen length (Fig. 1e).

### Comparison to other fossil and extant calmanostracans using ratios

**Fig. 3:**
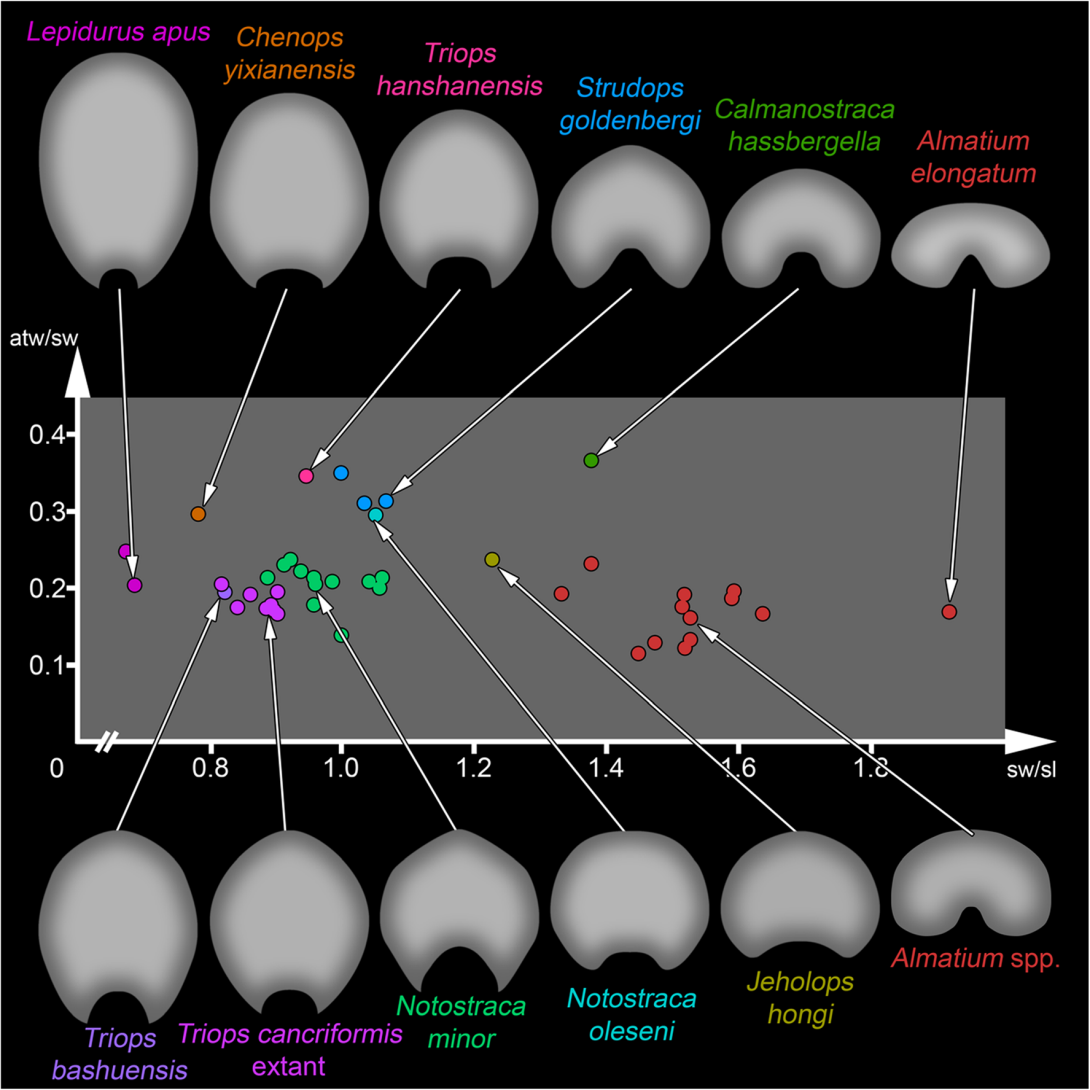
Anterior trunk width/shield width vs. shield width/shield length ratios in *Notostraca oleseni* and other extinct and living calmanostracans: Morphospace analysis using ratios. Overview of used material is given in Table 1

Plotting the ratios of anterior trunk width/shield width vs. shield width/shield length results in a plot with rather distinct clusters (Fig. 3). Representatives of Kazacharthra, in this case representatives of *Almatium gusevi*, *Almatium gobiense* and *Almatium elongatum* (here termed *Almatium* spp.) plot on the right side. All these have very broad and rather short shields, with *A. elongatum* possessing the broadest shield. The specimen with supposed kazacharthran affinities described by Wagner et al. [58] has a slightly more elongate shield, but which is still quite broad, in consequence also plotting right but not as far as most specimens of *Almatium*. In contrast to representatives of *Almatium*, which all possess a similarly wide anterior trunk in relation to the shield width, the specimen of Wagner et al. [58] has a broader anterior trunk. Therefore, the specimen plots above the representatives of *Almatium* on the scatter plot.

*Jeholops hongi* plots at the center. The shield is slightly broader than long and the anterior trunk has one fifth of the width of the shield. Hence it plots also close to specimens of *Almatium*.

One distinct cluster further to the left is formed by representatives of the modern species *T. cancriformis* and 200-million-year-old representatives of *Notostraca minor.* Still both species are separated and do not intermix in the plot. *N. minor* has a more roundish shield than *T. cancriformis*, which possesses a slightly longer than wide shield; therefore *N. minor* plots further right, *T. cancriformis* further left. The trunk width in relation to the shield width is nearly identical in these two groups. *Triops bashuensis* plots close to *T. cancriformis*, indicating a similar shield and anterior trunk shape.

Above this cluster are *Chenops yixianensis*, *Triops hanshanensis*, *Strudops goldenbergi* and the new fossil specimen. The new fossil possesses a roundish shield shape with the anterior trunk as broad as one third of the shield width. *S*. *goldenbergi* plots quite close to our specimen. The shield of *S*. *goldenbergi* also is quite roundish, but details of the shape are a bit different (see discussion below). *T*. *hanshanensis* plots left to the specimens of *S*. *goldenbergi* and the new fossil. The shield of *T*. *hanshanensis* is more elongate, has an elliptical shape and the anterior trunk is slightly broader. *C*. *yixianensis* possesses an anterior trunk with a similar width in relation to the shield width as does *S*. *goldenbergi* and our specimen, but the shield is more elongate. The elliptical shape of the shield is even stronger drawn out in anterior-posterior axis than in *T*. *hanshanensis*.

Representatives of *Lepidurus* form a cluster at the left edge of the scatter plot. The shield is one and a half times longer than wide. The shield is four to five times wider than the anterior trunk.

### PCA of size corrected data using Burnaby-Back-Projection

**Fig. 4:**
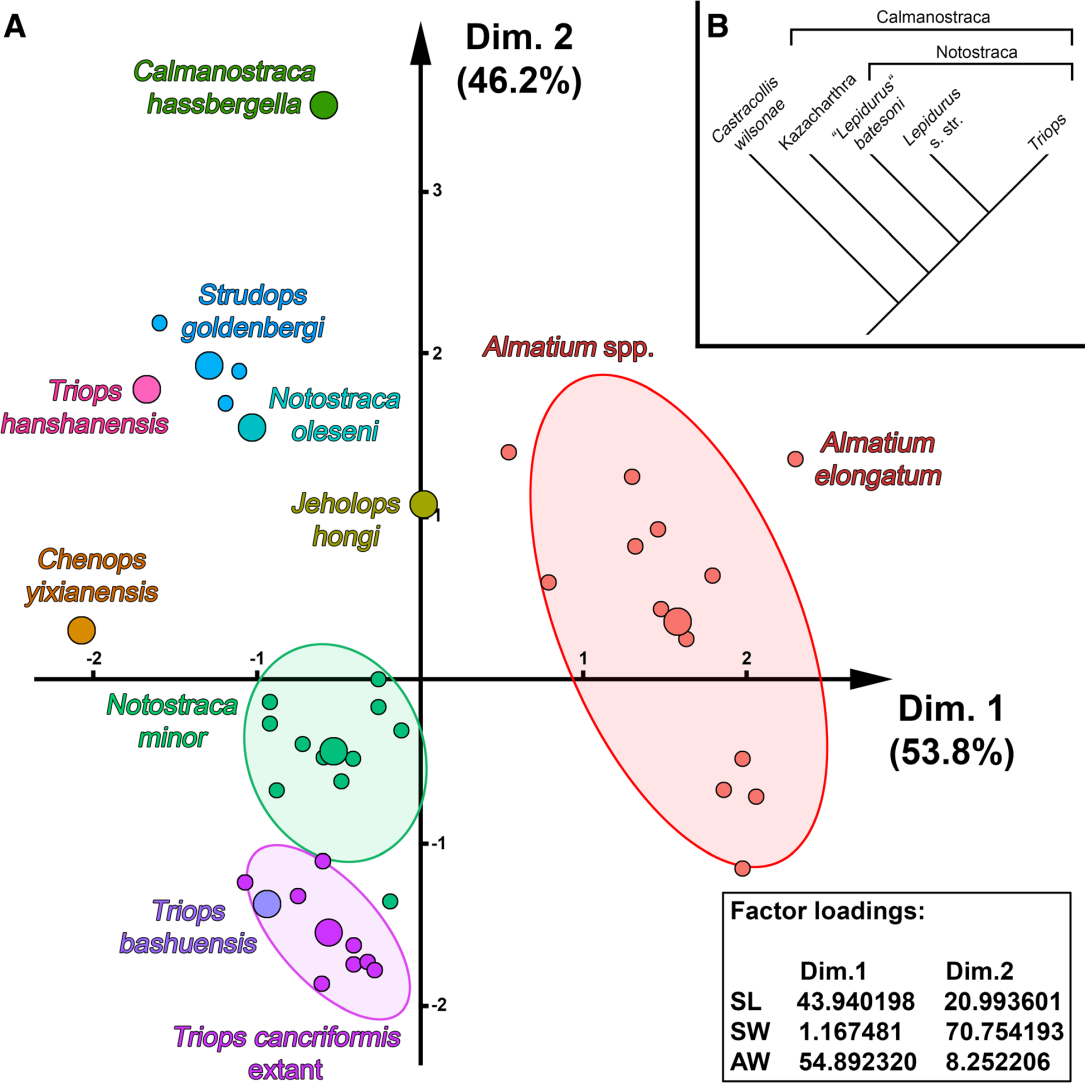
Morphospace using Burnaby-Back-Projection and its resulting implications: **a** Morphospace using Burnaby-Size-Correction and PCA, small circles represent included specimens, large circles represent the center calculated for the respective species, ellipses indicate a 75% confidence interval; **b** Simplified phylogenetic tree of Calmanostraca. *Castracollis wilsonae* was used to root the tree. Kazacharthra as sister group to Notostraca. A distinction is made between *Lepidurus batesoni* and *Lepidurus* s. str. (including all other species of *Lepidurus*) because the first one does not show a modified and elongated first thoracic appendage in contrast to *Lepidurus* s. str. and *Triops*

The PCA using the Burnaby-Size-Correction data displays the data in a different way (Fig. 4a). Dimension 1 (x-axis) represents 53.8% of the variance, dimension 2 (y-axis) 46.2%. About 55% of the variance of dimension 1 originate from the anterior trunk width, about 44% from the shield length and only about 1% from the shield width. About 70% of the variance of dimension 2 originate from the shield width, the rest from shield length and anterior trunk width (Table 2). The new fossil is again located close to *Strudops goldenbergi*. *Chenops yixianensis*, *Jeholops hongi* and *Triops hanshanensis* also plot in close distance. Three additional clusters are formed. The two clusters formed by the representatives of *Notostraca minor* and representatives of extant *Triops cancriformis* plot in close distance to each other while the distinct cluster formed by representatives of *Almatium* spp. plots in a different area of the morphospace. These three clusters are indicated by ellipses with a 75% confidence interval.

**Table 2:** Factor loadings of PCA

	Dim.1	Dim.2	Dim.3
Shield length	43.940198	20.993601	35.06620
Shield width	1.167481	70.754193	28.07833
Anterior trunk width	54.892320	8.252206	36.85547

## Discussion

### Comparison of two methods: Ratios vs. Burnaby-Size-Correction

The herein presented study explored two different methods of size correction when plotting data. The first method to perform a size correction is by calculating ratios and plotting these ratios against each other (Fig. 3). However, as mentioned above, the questions of using ratios for size correction and whether ratios eliminate the effect of body size satisfactorily have been hotly debated in the past [1, 2, 5, 25]. Therefore, we also performed a Burnaby-Size-Correction using the Burnaby-Back-Projection and calculated a PCA with these size-corrected data (Fig. 4a). When comparing both plots, it becomes apparent that, besides both displaying the data in different ways, they are in fact very similar. The size of the clusters, but also the position of different clusters to each other, is nearly the same. For example, *Strudops goldenbergi* plots in relatively close distance to *Triops hanshanensis* and *Jeholops hongi*. This is true for both plots. Hence ratios seem to provide quite reliable results, at least in this case. To our personal experience it seems that using ratios seems to work out quite well when dealing with more or less roundish structures (in this case the shield). If the structures are more elongated and slender, the results of the Burnaby-Size-Correction and of the ratio-based method begin to deviate from each other. In cases with roundish structures, ratios seemingly represent a suitable and especially easy approach to eliminate size. Ratios also provide one advantage over the Burnaby-Size-Correction: the original data do not need to be to scale. This might sometimes be favorable, especially when working with data from the literature, because not all authors do provide scale bars. It was, for example, not possible to use published images of representatives of *Lepidurus* for the Burnaby-Size-Correction as no scales were provided in the publications.

### Intra- vs. interspecific variation

Linder [36] and also Trusheim [55] claimed that shield shape varies widely within Notostraca. Longhurst [38] reinforced this by furthermore claiming that the shield shape is taxonomically useless. Hence, in his view shield shape should not be used for species delimitation.

Quite on the contrary, our plots (Figs. 3, 4a) do show a relatively small intraspecific variation, meaning that representatives of one species do plot in close distance to each other, forming distinct clusters. This is the case for representatives of the extant species *Triops cancriformis*, but also for the fossil species *Notostraca minor* and *Strudops goldenbergi*.

We feel it is important to highlight the clusters formed by *Triops cancriformis* and *Notostraca minor* explicitly*.* The specimens measured of these two species comprised several different ontogenetic stages, which means that these clusters are not only created by measurements performed on exclusively adult specimens, but also on several juvenile specimens. By including different ontogenetic stages these clusters contain ontogenetic information, additionally indicating morphological changes throughout ontogeny. Nonetheless, both species, *Triops cancriformis* and *Notostraca minor,* form very distinct clusters, implying a relatively small intraspecific variation, at least concerning the characters measured in this study.

All representatives of *Almatium* form a relatively distinct cluster, although being larger than the other clusters. This cluster is not only formed by specimens of one supposed species, but of supposedly several species (although the differentiation of the species is debatable). Hence the larger size of the cluster is to be expected.

We therefore suggest that the intraspecific variation of the shield shape is significantly smaller than the interspecific variation. Both intra- and interspecific variation are displayed in the generated morphospaces (Figs. 3, 4a). If the shield shape is highly variable within one species, the clusters should be much larger; intermixing with each other would also obscure the interspecific variation. This is not the case. Therefore, shield shape and also the herein generated morphospaces can contribute to species delimitation.

### Calmanostracan phylogeny

A major problem for understanding the evolutionary history of Calmanostraca is the general focus on a taxonomic, typological treatment of specimens without a strict comparative, phylogenetic one (but see e.g. [59]). It seems generally assumed that within Calmanostraca the exclusively fossil group Kazacharthra is the sister group of Notostraca (see e.g. [46, 59]). Notostraca then should comprise the two extant ingroups *Triops* and *Lepidurus*. Taking only this into account would be simple.

Yet, when it comes to include other fossil specimens this “simple” system becomes rather chaotic. Lagebro et al. [34] presented a phylogenetic reconstruction that resolved representatives of Kazacharthra in a polytomy together with *Jeholops hongi*, “*Triops” minor* and *Strudops goldenbergi* (“*Triops*” *minor* in this case is used to address the fossils Trusheim [55] described and named *Triops cancriformis minor*. Here we address this species as *Notostraca minor*; see [57] for a discussion). Lagebro et al. [34] treat this group of species together with modern representatives of *Triops* and *Lepidurus* as ‘Notostraca’. This implies that Notostraca becomes synonymous to Calmanostraca and that *Strudops goldenbergi* is the earliest representative of Notostraca [34].

Hegna and Ren [24] discussed whether Notostraca should be restricted to extant notostracan groups (*Triops* and *Lepidurus*), or should be used for all species with a notostracan-type shield (including extinct species). This question remains unresolved. Hegna and Ren [24] included both species described by them (*Chenops yixianensis* and *Jeholops hongi* ) into Notostraca, although stating that *Jeholops hongi* does show some kazacharthran affinities. Hence both Lagebro et al. [34] as well as Hegna and Ren [24] mentioned some indications that Kazacharthra is in fact an ingroup of Notostraca, making the latter a synonym of Calmanostraca. For the moment, we simply follow the preliminary strategy to treat clear representatives of Kazacharthra (in this case all representatives of *Almatium* spp.) as kazacharthrans, and all others as notostracans in the following (simplified in Fig. 4b). This is also in line with Walossek [59], who provided clear autapomorphies for both Notostraca and Kazacharthra and a list of differences between representatives of the two groups, including, e.g., number of thoracic segments, appendage morphology and telson shape.

Although the new specimen looks immediately “*Triops*-ish” its morphology requires further discussion. With its roundish shield it plots somewhere in between the kazacharthran representatives and the extant representatives of Notostraca, *Triops cancriformis* and *Lepidurus apus*. All kazacharthran species have a relatively broad and rather short shield, whilst extant notostracan species possess rather elongate shield forms. The area between these two groups is occupied by extinct notostracan species, such as *Notostraca minor*, *Triops hanshanensis*, *Strudops goldenbergi* and *Jeholops hongi*. The new specimen hence plots in an area where exclusively notostracan representatives plot, i.e. that have the notostracan-type shield shape. Additionally, a position in Notostraca is supported by details of the ventral morphology. Kazacharthra only possess a series of anterior thoracopods, possibly 11 pairs [59]. The new specimen described herein possesses a series of anterior and also a series of posterior thoracopods (Fig. 2d), resulting in a considerably higher number of thoracopod pairs. The preservation of the new specimen did not allow to check for epipods, a typical notostracan feature in contrast to Kazacharthra, as the proximal parts of the limbs are not preserved. A further feature of kazacharthrans is their telson with bulged lateral sides. This differs markedly from the square-shaped telson with postero-lateral notches (attachment site of the furcal rami) of the new specimen, typically known from extant and extinct notostracans. Therefore, we interpret the new specimen as a representative of Notostraca, and not Kazacharthra.

As mentioned above, depending on the phylogenetic position of the fossil species *Strudops goldenbergi* and/or *Notostraca minor*, Notostraca could become synonymous to Calmanostraca. The herein described specimen would (most likely) still be a representative of Notostraca but this name would refer to a different node.

### Comparison to *Strudops goldenbergi*

The new specimen plots close to *Strudops goldenbergi*. Therefore, we will discuss differences but also similarities between *S*. *goldenbergi* and the herein described specimen exploring whether these represent two different species. *S. goldenbergi* was collected from the Strud locality in southern Belgium, Late Famennian (Upper Devonian, 372–358 million years ago) [13, 34]. The Strud locality is separated by quite a distance from the Yixian Formation in modern-day China, as has already been the case in the Cretaceous, representing a significant geographic separation of *S. goldenbergi* and the new fossil.

The geological differences are also significant. *Strudops goldenbergi* is about 240 million years older than the new fossil. Morphological stasis has been claimed to be a major factor with quite some impact within Notostraca [28, 29, 38, 39, 56]. Hence, one could argue that separation by time should not be taken into account. More recent studies, however, showed that morphology might not be that static and that significant morphological differences can be found between notostracan species, set apart by some hundreds of million years [16, 34, 57].

Furthermore, the herein presented plots are created by only three measurements; hence only few morphological features are represented in these plots (in this case shield length, shield width and trunk width). Specimens might plot close to each other, indicating a closer morphological similarity. Yet this similarity would only take few morphological characters into account. This does not represent a major problem, as obviously most of the accepted species are well separated. Still we need to be aware of this. Measurements and plots represent first, simple tools to generally distinguish between different and similar morphologies, but further morphological differences need to be compared separately. Hence, in the following we will outline distinct morphological differences between *S. goldenbergi* (as described by Lagebro et al. [34] and updated by Gueriau et al. [17]) and the new fossil.

The first difference is the overall size. The average body length of *S*. *goldenbergi* from the anterior margin of the shield to the posterior margin of the telson is 9.5 mm (7.9–13.5 mm, *n*=6). The new fossil has a body length (again from the anterior margin of the shield to the posterior margin of the telson) of 46 mm, so is about 5 times as long as *S*. *goldenbergi*. This also applies for the shield. The shield of *S*. *goldenbergi* is in average 5.1 mm long (2.9 – 7.9 mm, n=6) and 6.5 mm wide (4.8–8.3 mm, *n*=7). Yet, this difference could be attributed to ontogenetic and not species differences.

A similarly possible ontogenetic difference is the trunk subdivision. The anterior trunk of *S*. *goldenbergi* is rather broad similar to that of the new fossil (one reason why they plot close to each other). Lagebro et al. [34] estimated the number of abdominal segments with 6–15. Counting abdominal segments on the new fossil is difficult as only the six posteriormost segments are clearly distinguishable. Still, even when carefully extrapolating the segments from this starting point on towards anteriorly the number of abdominal segments seems to be higher than 15 (estimation based on visible posterior segments: around 20 segments). The higher number of trunk segments could on a first glimpse also be interpreted as an ontogenetic difference. Besides difference in number, the individual segments in *S. goldenbergi* appear more elongated in anterior-posterior axis. This would demand for an ontogenetic shape change during ontogeny, which makes an ontogenetic interpretation at least less likely.

*Strudops goldenbergi* and the new fossil both have a subfrontal plate. Yet in *S*. *goldenbergi* this plate is very broad and much more prominent than in the new fossil. In both species, a dorsal carina or mandibular bulge are not apparent.

Further differences can be recognised at the posterior notch of the shield. The posterior margin of the shield of *S*. *goldenbergi* is stronger bent anteriorly (described as being bell-shaped with a smooth margin by Lagebro et al. [34]) than in the new fossil. The corners of the notch of *S*. *goldenbergi* are therefore more strongly drawn out but not pointed. (Lagebro et al. [34] described the corners of the notch as being subangular). The new specimen possesses more rounded corners of the notch. In addition, the anterior tip of the shield of *S*. *goldenbergi* is narrower and a little bit more pointed than in the herein described fossil, in which the tip of the shield is more roundish.

Differences regarding the head region cannot be discussed as parts of the head region of the new specimen are not preserved. Gueriau et al. [17] provided an updated description of *Strudops goldenbergi*, mainly focusing on one newly discovered specimen also including some details on thoracopod morphology. They describe the 4^th^ and 5^th^ endites as well as the endopod as serrated, trapezoidal structures located at the distal tip of the thoracopods. Additionally, they were able to describe these endites also for the first thoracopod, which in this case are elongated and possess a rather “antenna-like” appearance. Limb differentiation regarding the first thoracopod, especially an elongation of the 4^th^ and 5^th^ endites is a typical feature of most modern-day notostracans ([17], their Fig. 4a-d, possibly also [34], their Fig. 3b-d, f). The herein described new fossil does not possess these elongated endites on the first thoracopod, but overall morphology of the following appendages regarding their images seems to be similar to the herein described specimen.

The telson of *S*. *goldenbergi* has a rectangular shape, slightly tapering towards posteriorly. It is slightly wider than long and bears two furcal rami. The new specimen has a square-shaped telson, which seems to be a little bit more elongate than that of *S*. *goldenbergi*. The distance between the attachment sites of the two furcal rami also seems to be slightly wider. In *S*. *goldenbergi* these rami seem to attach closer to each other.

The new specimen shows an overall morphology not yet known. We therefore interpret the new specimen from the Yixian Formation as a new species of Notostraca. Besides *Chenops yixianensis* and *Jeholops hongi* the new fossil possibly represents the third notostracan species from this formation. Despite showing a roundish shield, a morphology known from *Strudops goldenbergi* from the Devonian period, several morphological differences indicate that *S. goldenbergi* and the herein described specimen should be interpreted as two separate species.

### Taxonomic treatment of the new fossil

Euarthropoda [60]

Crustacea *sensu lato* sensu [52] (amended [22])

Eucrustacea sensu [59]

Branchiopoda [35]

Calmanostraca [54]

Notostraca [49]

*Notostraca oleseni* sp. nov.

### Derivation of name

In honor of Jørgen Olesen, Copenhagen (Denmark), for his important contributions to several crustacean groups, especially branchiopods.

### Material

The material consists of only the holotype SMNS 70488

### Diagnosis

Notostracan crustacean with a roundish shield, ratio of shield width/shield length about 1.05; shield covering 65% of the main body (excluding furcal rami); shield with a posterior notch with rounded lateral corners; head region followed by anterior trunk region bearing anterior thoracopods, one pair of appendages per segment, and middle trunk region bearing a series of posterior thoracopods, several pairs of appendages per segment; segmented posterior trunk followed by a subtrapezoidal telson with two elongated multiannulated furcal rami.

### Differential diagnosis

Shield shape similar to that of *Strudops goldenbergi* regarding ratio of shield width/shield length, but posterior margin of the shield of *S*. *goldenbergi* stronger notched; corners of the posterior notch of *S*. *goldenbergi* stronger drawn out, but not pointed, while in *Notostraca oleseni* the corners of the notch are more rounded; anterior tip of the shield of *S*. *goldenbergi* narrower and slightly more pointed, while *N. oleseni* possesses a more roundish tip of the shield.

### Amendment to “A fossil crustacean from the Upper Triassic of southern Germany with kazacharthran affinities” [58]

Based on the herein performed measurements we can additionally delimitate the specimen described by Wagner et al. [58] from other species. Wagner et al. [58] did not name their specimen as they did not have a criterion for species demarcation. Measurements on shield and anterior trunk now provide a differential diagnosis which clearly shows that the specimen plots in an area which is not occupied by any other calmanostracan representative. It possesses a shield and anterior trunk morphology that demarcates it from the other forms. Hence it seems unlikely that the specimen represents one of the known species and therefore should formally receive a name.

Additionally, measurements show that the specimen most likely is closely related to Kazacharthra following our interpretation of Hegna & Ren [24]. The specimen has a shield shape known from kazacharthran representatives, but is differs from all other known kazacharthran species in its large anterior trunk width. However, phylogenetic relationships within Kazacharthra and especially, as discussed above, within Calmanostraca are not yet resolved. We do not want to add up to this problem and therefore suggest to use the supra-species name (as genus substitute) Calmanostraca, resulting in *Calmanostraca hassbergella* sp. nov.

Furthermore, we want to correct a scale bar error in Wagner et al. [58]. Figures 1 and 2 do not give the correct scale. The scale bar in Figs. 1a, c, d, 2a, b corresponds to 2.5 mm, in Fig. 1b, e, 2d to 5.0 mm, in Fig. 2c, e to 1.0 mm.

### Taxonomic treatment of Calmanostraca hassbergella

Euarthropoda [60]

Crustacea *sensu lato* sensu [52] (amended [22])

Eucrustacea sensu [59]

Branchiopoda [35]

Calmanostraca [54]

*Calmanostraca hassbergella* sp. nov.

- 2018 fossil crustacean from the upper Triassic of southern Germany with kazacharthran affinities – Wagner et al., p. 57
- 2018 specimen with kazacharthran-related traits – Wagner et al., pp. 57, 58
- 2018 kazacharthran-like specimen – Wagner et al., pp. 57, 59, Figs. 1 a–c, 2
- 2018 specimen SMTE 5930-2-12 – Wagner et al., pp. 58, 60
- 2018 specimen 1 – Wagner et al., pp. 58 – 62, Figs. 1 a–c, 2

### Derivation of name

Named after the Hassberge Formation, Upper Triassic (Carnian, ca. 237–227 Ma) of Lower Franconia, southern Germany, near Würzburg (“Coburger Sandstein”) and to highlight its importance to regional palaeontology. Numerous notostracan and spinicaudatan branchiopod crustaceans, as well as fishes, some insects and plant remains are preserved in this formation.

### Holotype

Collection number: SMTE 5930-2-12; stored in the Museum Terra Triassica in Euerdorf (Germany).

### Diagnosis

Calmanostracan crustacean with a broad shield, about 1.5 times as wide as long, with a posterior notch with rounded lateral corners; anterior trunk with a width to length ratio of 1/5 with at least 29 segments; posterior telson roughly square-shaped with bulged lateral sides.

## Conclusions

1. *Notostraca oleseni* represents a new notostracan species from the Yixian Formation (Late Jurassic or Early Cretaceous). Its overall morphology and also morphological details set it apart from other notostracan species known so far. Therefore, after *Chenops yixianensis* and *Jeholops hongi* it represents the third notostracan species described from the Yixian Formation.
2. The phylogenetic position of *Notostraca oleseni* remains unclear, as phylogenetic relationships within Calmanostraca are not yet satisfactorily resolved. Therefore, we use the next reliable node (Notostraca) in the phylogenetic tree as a supra-species name (“genus name”) to name the new species *Notostraca oleseni*. More studies would be needed to resolve the phylogenetic relationships within Calmanostraca and the position of *Notostraca oleseni* on this tree.
3. The herein presented study provides a tool to demarcate the fossil specimen described by Wagner et al. [58] from other calmanostracan species. The original description is amended and the species is named *Calmanostraca hassbergella*.
4. Calmanostracan representatives appear to show greater morphological diversity of shield forms than previously thought, ranging from broad and rather short shields in kazacharthrans to more slender and elongated forms in representatives of *Lepidurus*. Also the width of the anterior trunk seems to be highly variable in comparison to the shield width. Calmanostraca, a group that is often said to be strongly consistent in its morphology and often is reduced to its modern-day representative *Triops cancrifomis* seems to be much more diverse than commonly expected. Especially extinct forms add up to this and highlight the species richness and morphological diversity of the group Calmanostraca.
5. For a better understanding of the morphological diversity within Calmanostraca and the phylogeny and evolution of the group a larger-scale study will be needed, which goes beyond the scope of this study. This would on the one side include screening the literature for more publications presenting calmanostracan specimens, especially also in older and non-English literature (e.g. from the former Soviet Union and China). One example would be the publication by Yang & Hong [62] which includes calmanostracan specimens from the Upper Jurassic Dabeigou Formation, for which the species *Weichangiops triangularis*, *Weichangiops rotundus* and *Brachygastriops xinboensis* have been erected. On the other side, museum collections need to be checked on a larger scale for still neglected specimens. Also attempts to include less well preserved specimens need to be undertaken. The preservation of these fossils is challenging for taking measurements, but carefully dealing with such problems will show the capabilities and restrictions of the herein presented method.

## Data Availability

Please contact author for data request.
